# A feasibility randomised controlled trial with an embedded qualitative evaluation of perinatal emotional skills groups for women with borderline personality disorder: protocol for the EASE study

**DOI:** 10.1186/s40814-022-01177-y

**Published:** 2022-09-23

**Authors:** Paul Moran, Debra Bick, Lucy Biddle, Belinda Borries, Rebecca Kandiyali, Janice Rigby, Penny Seume, Vaneeta Sadhnani, Nadine Smith, Michaela Swales, Nicholas Turner

**Affiliations:** 1 Centre for Academic Mental Health, Population Health Sciences Department, Bristol Medical School, University of Bristol, Bristol, UK; 2 Warwick Clinical Trials Unit, Warwick Medical School, University of Warwick, Coventry, UK; 3 Population Health Sciences Department, Bristol Medical School, University of Bristol, Bristol, UK; 4 Specialist Community Perinatal Mental Health Service, Avon & Wiltshire Mental Health Partnership NHS Trust, Bristol, UK; 5 Centre for Health Economics, Warwick Clinical Trials Unit, University of Warwick, Coventry, UK; 6 Channi Kumar Mother and Baby Unit, Bethlem Royal Hospital, South London and Maudsley NHS Foundation Trust, London, UK; 7 Patient and Public Involvement and Engagement Lead, London, UK; 8 North Wales Clinical psychology Programme, Bangor University, Bath, UK

**Keywords:** Borderline personality disorder, Perinatal mental health, Psychological treatment, Clinical trial, Qualitative study

## Abstract

**Background:**

Borderline personality disorder (BPD) is a severe mental disorder characterised by emotional instability, impaired interpersonal functioning and an increased risk of suicide. There is no clear evidence about how best to help women with BPD during the perinatal period. Perinatal Emotional Skills Groups (ESGs) consist of 12 group sessions, focussing on core skills in emotion regulation, interpersonal effectiveness, distress tolerance and mindfulness and how these skills can best be utilised during the perinatal period. Prior observational research has shown that perinatal ESGs may help women with BPD. We set out to test the feasibility of conducting a randomised controlled trial to investigate the clinical effectiveness of perinatal ESGs.

**Methods:**

A two-arm, parallel-group, feasibility randomised controlled trial of Perinatal ESGs in addition to Treatment as Usual (TAU) versus TAU for women aged over 18 years, who are likely to have a diagnosis of BPD and are either pregnant or are within 12 months of having a live birth. We will exclude women who have a co-existing organic, psychotic mental disorder or substance use dependence syndrome; those with cognitive or language difficulties that would preclude them from consenting or participating in study procedures; those judged to pose an acute risk to their baby and those requiring admission to a mother and baby unit. After consenting to participation and completing screening assessments, eligible individuals will be randomly allocated, on a 1:1 ratio, to either ESGs + TAU or to TAU. Randomisation will be stratified according to recruitment centre.

Feasibility outcomes will be the proportion of participants: (1) consenting; (2) completing baseline measures and randomised; (3) completing the intervention and (4) completing follow-up assessments. All study participants will complete a battery of self-report measures at 2 and 4 months post-randomisation. A nested qualitative study will examine participants’ and therapists’ experiences of the trial and the intervention.

**Discussion:**

Evidence is lacking about how to help women with BPD during the perinatal period. Perinatal ESGs are a promising intervention and if they prove to be an effective adjunct to usual care, a large population of vulnerable women and their children could experience substantial health gains.

**Trial registration:**

ISRCTN80470632.

## Background

### Borderline personality disorder

Borderline personality disorder (BPD) is a severe mental disorder characterised by emotional instability and impaired interpersonal functioning [1]. People with BPD often experience anxiety, depression and self-harm and the suicide rate among people with the condition is fifty times higher than in the general population [2]. Typically, people diagnosed with BPD experience rapidly shifting emotions, have unstable relationships and their behaviour is impulsive. Considering these symptoms, the perinatal period can pose particularly difficult challenges for women with BPD, who may struggle to adapt to the demands of parenting and are more likely to experience adverse pregnancy outcomes [3]. Furthermore, their children are at increased risk of psychological problems and of being taken into care [4–6]. Pregnancy and the postnatal period present a unique opportunity for helping women with BPD, because there are multiple contacts with health professionals, as well as uniquely timed motivation from both the mother and health professionals to intervene, around the birth of a baby. At present, however, there is no clear evidence about how to help women with BPD effectively during the perinatal period [7].

### The treatment of borderline personality disorder during the perinatal period

Clinical guidelines recommend that people diagnosed with BPD should receive evidence-based psychological treatments [8, 9], including dialectical behaviour therapy (DBT)—a complex intervention that combines individual and group-based therapy. A Cochrane review of psychological therapies for people with BPD identified twenty-four randomised controlled trials (RCTs) of dialectical behaviour therapy (DBT) or modified DBT, yet none were focused on women in pregnancy and/or the post-natal period [10]. The NICE Guideline on Antenatal and Postnatal Mental Health [11] highlighted the lack of research into personality disorder during pregnancy and the postnatal period. It called for research to determine effective and cost-effective interventions for women with personality disorders in the perinatal period [12]. We conducted a systematic search for RCTs relating to the management of BPD during the perinatal period. Three ongoing studies of DBT for people with BPD were found by searching WHO’s International Clinical Trials Registry Platform (http://apps.who.int/trialsearch/); one is a non-randomised study; the remaining two studies are trials of standard DBT treatment being undertaken in the Netherlands (ID: NL7699) and in Australia (ACTRN12618001687280). Although these trials do not exclude women in the perinatal phase, neither trial is evaluating the effectiveness of ESGs for women with BPD during the perinatal period.

### Emotional skills groups

DBT can improve the mental health of people with BPD [13] by teaching people how to cope healthily with stress and improve their relationships. However, DBT is a lengthy and expensive treatment lasting up to 18 months and within public health systems, access to DBT is limited. DBT includes both individual and group work; the group work consists of facilitated, skills-based groups, called emotional skills groups (ESGs). ESGs teach individuals how to deal with difficult emotions and when delivered as a stand-alone intervention, they have been shown to help people with BPD [14, 15]. ESGs, therefore, have the potential to increase access to a key ingredient of DBT, with overall benefits in terms of cost effectiveness and wider dissemination within the NHS. However, we do not know whether ESGs, adapted for the perinatal period, are helpful for women with BPD. In this protocol, we describe a mixed methods randomised feasibility trial that aims to examine the feasibility of conducting a randomised trial to test the clinical and cost effectiveness of ESGs for women with BPD during the perinatal period. The study follows the guidelines for feasibility trials outlined by the SPIRIT 2013 Statement [16].

## Methods

### Aim

The aim of this study is to investigate whether it is feasible and acceptable to undertake a trial of the effectiveness and cost-effectiveness of perinatal emotional skills groups (ESGs) for women with BPD (in addition to standard care) compared with standard perinatal mental health care alone.

The specific objectives are the following:

1. To optimise methods for the identification of potential participants
2. To assess how many women accept the invitation to participate in the study
3. To determine whether the eligibility criteria are appropriate, too open, or too restrictive
4. To determine whether it is feasible and acceptable to randomise women with BPD to perinatal ESGs
5. To describe standard perinatal mental health care for women with BPD
6. To determine retention rates for treatment
7. To assess the completeness of follow-up data collection.
8. To assess the extent of clustering of outcome data to assist calculation of the sample size for a full-scale trial
9. To assess the acceptability and feasibility of the outcome and resource measures for a definitive trial
10. To produce a protocol for a definitive trial and economic evaluation.

### Design

The EASE study is a two-arm, parallel group, randomised controlled trial with a nested qualitative study, comparing perinatal ESGs in addition to standard perinatal mental health care, to standard perinatal mental health care only, for women diagnosed with BPD.

### Setting and participants

Study participants will be recruited from perinatal mental health services in two English mental health Trusts-South London & Maudsley NHS Foundation Trust in South East London and Avon & Wiltshire Partnership Mental Health Trust in South West England.

South London & Maudsley NHS Foundation Trust is the largest provider of NHS mental health services in the UK and covers four South London boroughs: Southwark, Lambeth, Lewisham and Croydon. The Trust serves a local population of 1.3 million people in South London and each year provides inpatient care for over 5000 people, and treats more than 40,000 patients in the community. Each of the four boroughs have high rates of diversity, population movement, drug use, crime and socio-economic deprivation [17].

Avon & Wiltshire Partnership Mental Health Trust provides secondary and specialist mental health services for a population of 1.8million people, over a large geographical area that includes South Gloucestershire, Bristol, Bath, North Somerset and Wiltshire. Each year, the Trust cares for approximately 75,000 adult service users, from a wide variety of backgrounds. Although the Trust covers large rural areas with relatively low deprivation, Bristol and North Somerset have deprivation ‘hot spots’ that are among the most deprived areas in the country [18].

### Participant eligibility, screening and consenting

**Fig. 1:**
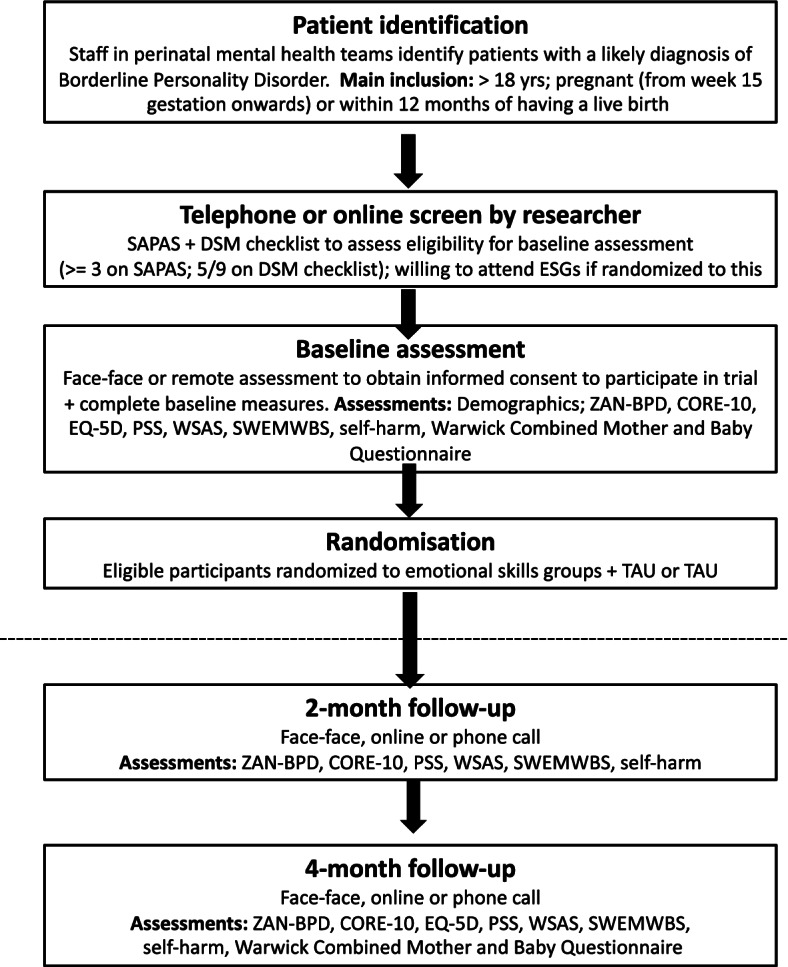
Study flow chart

Figure 1 outlines the key phases of the trial. We will first identify a key clinical contact in each participating team who will be asked to provide a list of all potential participants who meet the following inclusion criteria:

1. At least 18 years old
2. Likely to have a diagnosis of BPD
3. Either pregnant (from week 15 gestation onwards) or are within 12 months of having a live birth.

Women will be excluded if:

1. They have a current clinical diagnosis of a co-existing organic, psychotic mental disorder or substance use dependence syndrome
2. They have cognitive or language difficulties that would preclude subjects providing informed consent or compromise participation in study procedures
3. They pose an acute risk to their baby, as assessed by clinicians
4. They require admission to a mother and baby unit
5. They are unable to speak English with sufficient fluency to participate in study procedures

If the clinician thinks that an individual is likely to meet the eligibility criteria, they will approach them, provide verbal and written information about the study and determine whether they are agreeable to being contacted by a member of the research team with a view to assessing their eligibility for participation in the trial. This initial approach about the study will either be made face-to-face during a routine appointment, or over the telephone where basic information about the study will be given. Individuals will be given (or posted, if contacted by telephone) a copy of a Patient Information Sheet (PIS). The PIS explains that if the individual agrees for their details to be shared with the research team, then the initial step is a telephone or online screening interview. If the individual expresses an interest in the study, they will be asked if they would provide verbal consent to be contacted by a study researcher. The contact details and preferred method of contact will then be passed on to the researcher who will arrange a time to conduct a telephone or online interview (depending on the individual’s preference) with women to assess their eligibility for the study. Potential participants will be given at least 24 h after receiving the PIS and before being contacted by the researcher for an assessment of their eligibility.

### Screening assessment of potential participants

The researcher will first briefly explain the study, check that a PIS has been received and answer any questions the participant may have. With verbal consent, the researcher will then proceed with the screening assessment which will consist of three elements:

1. We will use the self-report Standardised Assessment of Personality Abbreviated Scale (SAPAS) [19] to check that potential participants have probable personality disorder. The SAPAS is a reliable, valid and acceptable scale for assessing personality-related difficulties. A score of three or more on the SAPAS correctly identifies 90% of people with DSM-IV personality disorder and has a sensitivity 0.94 and specificity 0.85 [19]. To be included in the study a potential participant will need to score 3 or more on the SAPAS.
2. The researcher will also complete a 15-item BPD checklist with the patient (which has been used in the UK Adult Psychiatric Morbidity Survey [20]), confirming the presence or absence of DSM symptoms of BPD. In keeping with international diagnostic guidelines [1], patients will need to positively endorse at least 5/9 of the symptom domains covered by the 15 items to be eligible for the trial.
3. The researcher will check whether the patient would be willing and able to receive emotional skills groups (face-to-face or online) if they were randomised to this.

If a woman meets the screening criteria, they will be offered a baseline assessment with the researcher. Those who are ineligible will be thanked for their time and informed of the reason(s) for this. Following the eligibility check and prior to commencing the baseline assessment, the researcher will obtain the written consent of all eligible women relating to their participation in the trial. The researcher will answer any further questions the individual may have, and re-check whether they are still willing and able to receive emotional skills groups if they were randomised to this. Consent will be obtained either via an online ‘e-consent’ method (if the baseline assessment is being conducted remotely) or via paper-based informed consent, if the researcher is meeting with the individual face-to-face. Individuals attending a face-to-face appointment will be given a copy of their written consent form to keep. Individuals completing the assessment remotely will receive an electronic copy of their e-consent form via email. A copy will also be sent to the patient’s GP.

Participants will be reminded that they are free to withdraw from the trial at any time without giving reasons and without prejudicing their further treatment. We will seek consent to use data collected up to the point of withdrawal, and this will be explained in the information sheet. In addition, in line with open access data requirements, information may also be used to support other research in the future and may be shared anonymously with other researchers. This will be explained in the information sheet. As part of the baseline consent procedure, individuals who give informed consent for trial participation will be asked to indicate whether they would be willing to be contacted about future-related research. Participants will be informed that to reimburse their time and effort, they will be offered £20 vouchers at the baseline, 2-month and 4-month follow-up assessments.

### Baseline assessment of participants

The baseline assessment will proceed once written consent has been obtained. The baseline assessment will last about 1 h and will take place face-to-face or online (depending on preference) with the participant completing online questionnaires on their own computer or mobile device, and the researcher providing support via telephone or videocall.

Participants will be asked to complete the following questionnaires (below) selected following discussion with women who have lived experience of using perinatal mental health services. The burden of collecting these measurements on participants will be assessed during the study:

#### Sociodemographic information

Data on participants’ relationship status, age, gender, ethnicity, highest education level attained, living arrangements and current employment status will be recorded. They will also be asked a question about whether they are in the antenatal or post-natal period.

#### Symptoms of borderline personality disorder

BPD symptoms will be assessed using the Zanarini Rating Scale for Borderline Personality Disorder Self-Report Scale (ZAN-BPD) [21]. The ZAN-BPD is a widely used measure of symptoms experienced by people diagnosed with BPD. The measure covers a 1-week time frame and each of the nine criteria for BPD is rated on a 5-point anchored rating scale of 0–4, giving a total range of scores between 0 and 36 with higher scores indicating poorer mental health. The ZAN-BPD has been used in previous trials of treatments for people diagnosed with BPD and is sensitive to change [22].

#### Psychological distress

Symptoms of psychological distress will be assessed using the 10-item Clinical Outcomes in Routine Evaluation (CORE-10) [23]. The CORE-10 is a brief measure of psychological distress derived from the larger CORE-OM, a well-established measure for evaluating psychological therapies in services in the UK [24]. The CORE-10 has displayed good psychometric properties in previous trial participants [25].

#### Health-related quality of life

This will be assessed using the EQ-5D-5L—a descriptive system and a visual analogue scale (VAS) for health-related quality of life states in adults. It has been shown to be sensitive to change among people with personality disorder [26].

#### Mental wellbeing

Mental wellbeing will be assessed using the Short Warwick Edinburgh Wellbeing Scale (SWEMWBS), a 7-item scale of mental well-being covering subjective well-being and psychological functioning. The seven statements are positively worded with five response categories from ‘none of the time’ to ‘all of the time’. SWEMWBS has shown high internal consistency in the UK population [27].

#### Social functioning

The Work and Social Adjustment Scale (WSAS) [28] is a simple, reliable and valid measure of impaired functioning that is sensitive to change. It consists of five items, with eight response categories per item, ranging from 0 ‘not at all impaired’ to 8, ‘very severely impaired’.

#### Parenting stress

The Parenting Stress Scale (PSS) [29] is an 18-item self-report measure of an individual’s feelings about positive and negative aspects of parenthood. It was developed with a view to assessing outcomes of parenting interventions across a wide age range of children. Possible scores range from 18 (low stress) to 90 (high stress). The PSS has good internal consistency and test-retest reliability [29].

#### Self-harming behaviour

Self-harming behaviour over the past week, will be measured using a single question: “Have you [in the past week] deliberately taken an overdose (e.g., of pills or other medication) or tried to harm yourself in some other way (such as cut yourself)?” [30]. Responses are rated 1—yes, once; 2—yes, more than once; or 3—no.

Arrangements for starting therapy will be discussed with those allocated to receive the intervention and these individuals will also be shown how to log onto the therapy platform. This discussion will take place during the baseline assessment if appropriate or, if necessary, a separate arrangement (for a later date) will be made with the participant to discuss therapy arrangements and login instructions.

### Interventions

Those in the active arm of the trial will be offered ESGs in addition to standard perinatal mental health care, while those in the control arm will continue to receive standard perinatal mental health.

### Perinatal emotional skills groups

Perinatal Emotional Skills Groups (ESGs) will be delivered as specified in the Maternal Emotional Wellbeing manual [31]. An uncontrolled evaluation of ESGs in a cohort of 21 women, found that there was strong evidence of a reduction in mental distress over the intervention period (Cohen’s *d* = 0.83; *p <* .001) and at the end of treatment, women reported substantial improvements in their ability to manage difficult emotions [31]. The intervention comprises up to 2 individual preparatory sessions, followed by 12 group sessions. The individual sessions last up to 90 min and subsequent group sessions last up to 2 h. Participants receiving ESGs will also continue to be cared for as usual by their perinatal mental health team.

**Fig. 2:**
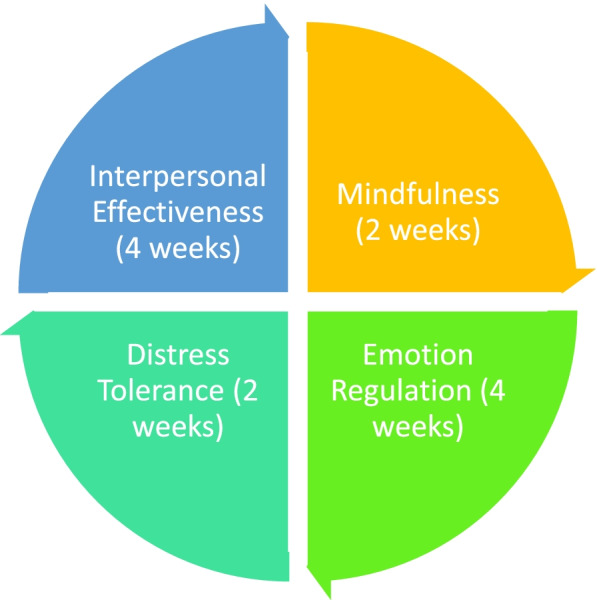
Over-arching content of ESGs

**Fig. 3:**
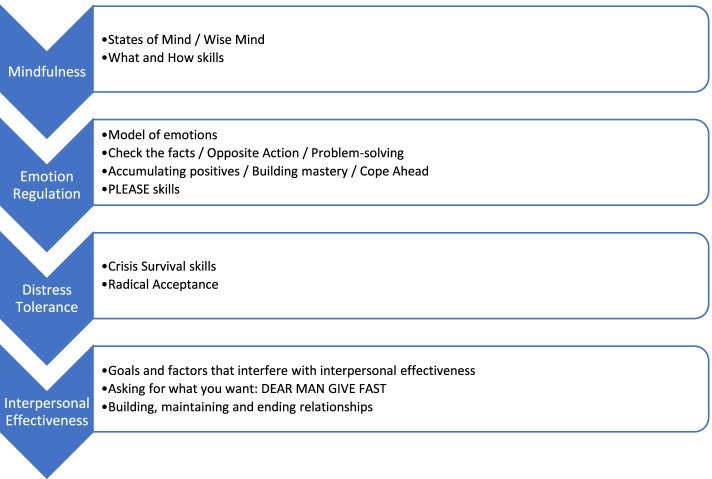
Content of each ESG module

The over-arching content of perinatal ESGs is displayed in Fig. 2 and the details of each module are displayed in Fig. 3. Groups usually treat up to 6 women and the groups are organised into four modules on emotion regulation, distress tolerance, mindfulness and interpersonal effectiveness (Fig. 2). These modules are focused on the acquisition of emotional skills and each session is supplemented with “Keeping Baby in Mind” teaching skills relevant to becoming a parent of a new child; these skills can be taught and practiced both prenatally, as well as postnatally. The groups will be run by a clinical psychologist working with a trained nurse or other qualified perinatal clinician.

At the end of treatment, the participant’s GP will be informed that they have completed therapy. The GP will also be informed if they withdraw from therapy or are discharged for non-attendance.

### Therapist training and supervision

Therapists will be mental health professionals with appropriate qualification and experience. All therapists will be employed by the local NHS Trusts, for the duration of the study. Therapists will receive training in ESGs 1 month before the start of treatment delivery. We will train up to 6 staff per site to deliver perinatal ESGs. Training will be provided during a two-day online workshop, led by co-author MS, who is an approved trainer. Four, half-day remotely conducted top-up sessions will also be conducted over the course of the study. We will also arrange regular online Microsoft Teams consultation meetings between centres, to promote sharing of good clinical practice through peer monitoring and encouragement.

During the study, following good clinical practice, therapists will receive weekly supervision from an experienced therapist in accordance with professional (British Association for Behavioural and Cognitive Psychotherapies) standards and local NHS practice.

### Attendance at ESG sessions

The EASE therapists will monitor the participant’s therapy attendance. Participants who miss three sessions in a row will be considered to have dropped out of treatment. A nested qualitative study will investigate participants’ views and experiences of ESGs, and help to identify reasons for completing or not completing treatment.

Participants who drop out from treatment will still be invited to complete follow-up assessments and, if applicable, to participate in a qualitative interview, unless they have explicitly indicated that they wish to withdraw from the study).

### Comparator

The comparator treatment will be standard perinatal mental health care, delivered on an individual basis, in accordance with current NICE and Royal College of Psychiatry guidelines [12]. It should consist of assessment, a written care plan and weekly reviews with a care coordinator. All study participants allocated to receive the comparator treatment will complete all the study assessments. Information on standard care will be gathered at 4 months post-randomisation.

### Outcomes

#### Feasibility outcomes

The main aim of this study is to demonstrate the feasibility of conducting a trial to evaluate the intervention, including the feasibility and acceptability of randomising the intervention during the perinatal period and the feasibility of outcome measure collection.

Feasibility of recruitment will be assessed by exploring:

1. The appropriateness of the eligibility criteria, as measured by the number of those referred to the trial over the study period, who meet the eligibility criteria.
2. The success of recruitment, as measured by the number of eligible patients who consent to participate in the trial over the study period, and the number of patients who decline to participate.
3. Retention rates, as measured by the number of participants who consent to participate that remain in the trial by 4-month follow-up.

Feasibility of the assessment battery will be assessed by measuring:

1. The number/proportion of participants with complete baseline data over the study period
2. The number/proportion of participants with complete follow-up data at 4 months follow-up.

Feasibility of the intervention will be assessed by measuring:

1. The number/proportion of participants attending all 12 sessions of treatment during the treatment phase of the study
2. The number/proportion of participants attending at least 9 sessions of treatment during the treatment phase of the study
3. The number/proportion of participants retained at the end of each module of treatment.

### Participant-centred outcome measures

As well as feasibility outcomes, data on participant-centred outcome measures proposed to be used in the main trial will also be collected. We will do this using a range of techniques according to participants’ preference including, online, telephone and paper questionnaires. This will allow exploration as to which techniques for data collection are most acceptable and feasible.

**Fig. 4:**
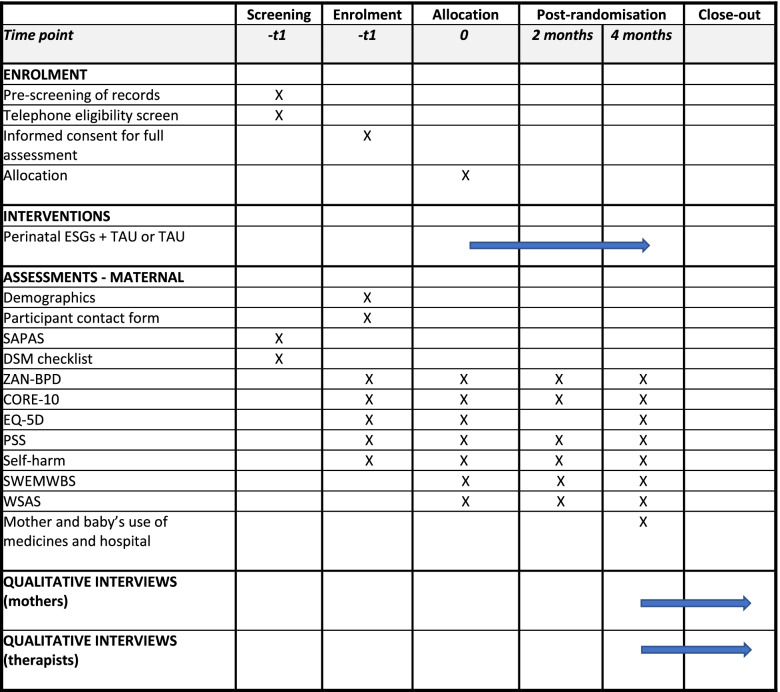
SPIRIT figure

The timing and sequence of all assessments are summarised in the SPIRIT figure (Fig. 4). All study participants will complete follow-up assessments at 2 and 4 months post-randomisation. At the 4-month data collection point, all the outcomes that were assessed at baseline will be reassessed. In addition, at 4-month follow-up, resource use will be collated by maternal self-report.

### Nested qualitative study

A qualitative study will be nested within the feasibility trial to help provide insight into participants’ and therapists’ experiences of the trial and the intervention. This will provide further information about the feasibility and acceptability of the intervention and future definitive trial design. After the completion of treatment, purposive samples of patients and staff will be invited to participate in a qualitative interview. All interviews will use a topic guide, will be audio-recorded using an digital recorder, and will be fully transcribed and analysed thematically. Consenting participants will be offered a £20 voucher to reimburse their time and effort for attending the qualitative interviews. The following groups will be interviewed:

1. Participants in the intervention and standard care arms.

These will be conducted at the completion of treatment with a purposive sample of between 10 and 15 participants. Maximum variation sampling will ensure that, where possible, women from different socioeconomic status, ethnicity and different levels of attendance at the intervention are selected. We will reflect upon sample requirements as data collection progresses. Questions will include views on the recruitment process, including screening and randomisation; motivation for joining the study; women in the intervention arm will be asked about aspects of the intervention they found most helpful and most challenging and all women will be asked about their experience of usual care; content and format of ESG sessions (e.g. length, online delivery); and feedback on the appropriateness of the measures being used, including burden of completion and preferred methods of administration.

2) Study decliners and participants who drop out of treatment

We will seek the views of up to 10 eligible women who, at some stage in the recruitment process, decide to decline participation or the views of participants who withdrew partway through. Interviews with these women will also allow us to explore their views about standard care. It is likely that participants who decline or drop out will be less willing to talk to the researchers, but we will attempt to capture their views, assuming they are willing to give consent. Questions will include reasons for declining or dropping out and whether their continuing participation in the study could have been better supported.

3) Staff interviews

Interviews with all practitioners and their supervisors will be carried out at the end of the intervention. These will focus on the ease of delivery, the value of training and supervision, as well as the acceptability, strengths and weaknesses of the intervention and the research process. The staff interviews will also seek staff insights into the feasibility of rolling online ESGs out more widely across the NHS, including their views about the training and funding required to support this activity.

### Sample size

In keeping with recommendations for feasibility studies [32], we have not based plans for sample size on a power calculation. A pragmatic sample size of 48 will allow us to estimate, with a desirable degree of precision, the rate of recruitment and retention in a future phase III trial. In 2018, in the selected catchment areas covered by Bristol and London, there were a total of 24,697 live births (www.ons.gov.uk). Assuming a community prevalence of BPD of 2% [33], across the 2 centres, 494 women with BPD could have been eligible for recruitment. Our recruitment target of 48 women from a pool of 494 (9.7%) is a low recruitment target, but we have been cautious as people diagnosed with BPD can be harder to recruit to studies [34]. Notwithstanding, our collaborators in perinatal services, the third sector and our advisors with lived experience of personality disorder have all encouraged us to undertake this study. We therefore think that there will be significant interest in the research and that the recruitment rate will be higher. Assuming a conservative recruitment estimate of 9.7%, a sample size of 48, will give us a 95% confidence interval for recruitment of between 7.3% and 12.7%. The trial aims to recruit 48 participants over 12 months from 2 perinatal mental health services at 2 trial centres, with a target of approximately 4 participants randomised per month.

### Assignment of interventions

After consenting to participation and completing screening assessments, eligible individuals will be randomly allocated, on a 1:1 ratio, to either ESGs + TAU or to TAU. The randomisation sequence will be generated by the Research Electronic Data Capture Service (REDCap) at the University of Bristol (https://brtcclinical.bris.ac.uk/redcap/). Randomisation will be stratified by centre. Appropriate staff at all sites, as delegated by the PI, will be provided with log-in details for the secure online randomisation system. Throughout the study, the randomisation list will be encrypted and held with the Trial Coordinating Office to ensure that the study researchers remain blinded to treatment allocation. At the end of the study, the randomisation list will be unencrypted and placed in the Trial Master File.

### Blinding

It will not be possible to blind participants or the treating clinicians to the participant’s treatment allocation because of the nature of the intervention. The statistician and health economist will be blind to the trial condition throughout the feasibility trial. The Trial Manager and research assistants will not be blind to the group as they will also be responsible for participant recruitment and reminder telephone calls for completion of follow-up data.

### Adverse events

All adverse events will be assessed for seriousness, causality and expectedness by the Principal Investigator and will be recorded and reported from the point of randomisation until the 4-month follow-up assessment or the point of withdrawal from the study. Hospitalisations for elective treatment of a pre-existing condition will not be reported as serious adverse events.

### Data management

A web based electronic Case Report Forms system will be used to collect baseline and outcome data. Study data will be archived securely and destroyed after 10 years. No data analysis will be undertaken until databases are locked.

Trial staff will ensure that the participants’ anonymity is maintained through protective and secure handling and storage of patient information at the trial centres. Each study participant will be assigned a unique participant identification number at the start of the assessment process. This number will be written on all assessment forms, contemporaneous notes and the database used to record data on study participants.

All documents will be stored securely and made accessible only to trial staff and authorised personnel. A hard copy of a record sheet linking patient identity and the randomisation code for all participants will be kept at each site along with the Participant Contact Details Form. Both will be placed in the Investigator Site File, in a locked filing cabinet, separate from the paper CRFs and other documents (e.g. contemporaneous notes) relating to a participant, which will be anonymised. Once the study is over, the site investigator will arrange the long-term storage (archiving) of all research data which will include the record sheet linking patient identity.

An electronic copy of participants’ contact details will be stored on a secure drive. At the end of the study, the electronic record sheet and the hard copy of the Participant Contact Details will be destroyed once interested participants have received a copy of the study results. Hard copies of trial allocation letters that are sent to participants and clinical teams will be stored in the Trial Master File and archived as evidence of the detailed trial procedures. Data from screening, baseline and follow-up assessments will be stored on a secure database and access will be restricted to members of the research team. Audio recordings and interview transcripts will be stored securely on a password protected server at the university co-ordinating centre. The contact details of therapists delivering the study intervention will be kept on an encrypted file at University of Bristol and will be deleted at the end of the study.

### Data analysis

Data will be analysed and reported following the CONSORT guidance extension to feasibility studies [35], including a CONSORT flow diagram and focussing principally on descriptive statistics of key feasibility parameters. We will assess the feasibility and acceptability of the trial design by calculating proportion (and 95% CIs) of participants: (1) consenting; (2) completing baseline measures and randomised; (3) completing the intervention—i.e. attending at least nine out of 12 sessions (75%) (4) completing follow-up assessments. Descriptive statistics for patient characteristics and outcomes will be reported overall and by treatment group; as means or medians with measures of dispersion for continuous data (as appropriate given the form of their distribution) and frequencies and percentages for categorical data. For participant outcomes, the proportion with complete data, for each outcome, will be reported in addition to descriptive statistics. The effect of treatment on outcomes will be estimated on an intention-to-treat basis and reported as difference in means between groups and associated confidence intervals only, since the study has not been powered for formal statistical hypothesis testing.

Data from the nested qualitative study will be analysed thematically using NVivo [36] to aid data management. The interview transcripts will be individually read and re-read, from which an initial coding framework will be developed. Independent double coding of a subgroup of transcripts will take place. Team members will meet to discuss the developing coding framework, to ensure that the emerging analysis is trustworthy and credible. This framework will be refined, with coded material regrouped, as new data from subsequent interviews are gathered and a deeper level of understanding is achieved. Descriptive accounts will be produced of main themes and constant comparison will be used to explore similarities and differences across groups of participants.

### Economic evaluation

Whilst it will not be possible to conduct a full economic evaluation, we will investigate the feasibility of undertaking a full economic evaluation. We will ask participants to complete a Mother and Baby Questionaire, to examine patterns of health and social care service use. Participants will also be asked to complete the EQ-5D-5L. We will identify the main cost drivers, by looking at the frequency and value of resources used, as well as any differences between arms. For economic outcomes, we will assess completeness and floor/ceiling effects in utilities at each time point with a view to assessing the suitability of the measure for a cost/QALY measure.

### Progression criteria

Our criteria for determining the success of the feasibility study are recruitment of at least 36 participants (75% of the target sample); uptake of ESGs by at least 75% of participants in the active arm of the trial and completion of 4-month follow-up assessments by 75% of the target sample. To determine the feasibility of calculating a cost analysis of health economics, we will record completion rates for the cost data and analyse them to determine what the cost-drivers are likely to be if proceeding to a full clinical trial.

### Ethics

Approval for the study has been given by Camden & Kings Cross Research Ethics Committee (Reference 21/LO/0833). The trial will be conducted in compliance with the principles of the Declaration of Helsinki, the principles of Good Clinical Practice, and all of the applicable regulatory requirements (UK data protection laws (meaning the Data Protection Act 1998 until 24 May 2018, and the European Union General Data Protection Regulation (GDPR) and applicable UK legislation that enshrines GDPR into UK law).

As part of the informed consent process, participants will be advised and provided guidance about confidentiality and the limits to it. Significant risk of future harm to self or their baby will be disclosed to their healthcare professional.

The trial may be prematurely discontinued by the Sponsor or Chief Investigator on the basis of new safety information or for other reasons given by the Trial Steering Committee, regulatory authority or ethics committee concerned. The trial may also be prematurely discontinued due to lack of recruitment or upon advice from the Trial Steering Committee. If the study is prematurely discontinued, active participants will be informed and no further participant data will be collected.

## Discussion

Women with BPD experience unique challenges during the perinatal period and currently there is a dearth of evidence about how best to help them during this important phase of the life course. This study will test the feasibility of using a randomised trial to examine the clinical and cost effectiveness of perinatal Emotional Skills Groups for women with BPD. Pre-trial non-randomised pilot work [31] has shown that perinatal ESGs are a promising intervention and if ESGs prove to be a clinically and cost-effective adjunct to usual care, a large population of vulnerable women would experience substantial health gains. Furthermore, given the inter-generational health risks associated with BPD [5, 37], enhancing the coping skills of women with BPD may also benefit the health of their children.

The trial has a number of strengths. We have adopted a pragmatic design, with broad inclusion criteria and a limited number of exclusion criteria. BPD often co-occurs with other mental health problems and we will therefore include women with BPD who have co-morbid depression, anxiety or substance use disorders. The latter category is particularly important as women with substance use problems are often excluded from BPD treatment trials. We decided to include pregnant and post-natal women in the trial for three reasons: first, the inclusion of both groups reflects the reality of perinatal mental health service delivery; second, treatment may offer benefits to women across the perinatal period; finally, prior work [17] shows that it is acceptable to run groups with both pregnant and post-natal women and that most women do not view pregnancy and the post-natal period as ‘separate’ parts of their maternity experience. The outcome domains have been carefully selected following consultation with women with lived experience of perinatal mental health services, as well as health care professionals. In addition, the collection of both qualitative and quantitative data will allow us to make a comprehensive assessment of the acceptability and feasibility of the study design.

There are also some limitations of the trial. In keeping with the approach adopted by other recent trials in the personality disorder field [38, 39], we have not included a detailed assessment of personality disorders. This is because such assessments are time-consuming to complete and this is not in keeping with the needs of a pragmatic study that will be conducted in a busy clinical setting. We will however be screening all potential participants with the SAPAS, which has good predictive validity [40–42]; these baseline data will provide useful information about the severity of personality disturbance. A further limitation is the restricted length of follow-up to 4 months. We have adopted this approach because our primary aim is to establish the feasibility of recruitment and randomisation. However, we acknowledge that BPD is a long-term condition, and a future definitive trial would benefit from having a longer-term follow-up of at least 12 months.

## Data Availability

Not applicable.

## Abbreviations

BPD: Borderline personality disorder
CORE-10: Ten-item Clinical Outcomes in Routine Evaluation Scale
CRF: Case Report Form
EQ-5D-5L: EuroQol Group 5-Dimensions 5-level
ESGs: Emotional Skills Groups
GDPR: General Data Protection Regulation
PIS: Participant Information Sheet
PSS: Parenting Stress Scale
SAPAS: Standardized Assessment of Personality-Abbreviated Scale
TAU: Treatment as usual
ZAN-BPD: Zanarini Rating Scale for Borderline Personality Disorder Self-Report Scale
SWEMWBS: Short Warwick Edinburgh Wellbeing Scale
WSAS: Work and Social Adjustment Scale
